# The latent structure of post-traumatic stress disorder among Arabic-speaking refugees receiving psychiatric treatment in Denmark

**DOI:** 10.1186/s12888-016-0936-0

**Published:** 2016-09-05

**Authors:** Erik Vindbjerg, Jessica Carlsson, Erik Lykke Mortensen, Ask Elklit, Guido Makransky

**Affiliations:** 1 Competence Centre for Transcultural Psychiatry, Mental Health Centre Ballerup, Copenhagen, Denmark; 2 Department of Psychology, University of Southern Denmark, Odense, Denmark; 3 Department of Public Health and Center for Healthy Aging, University of Copenhagen, Copenhagen, Denmark

**Keywords:** Cross-cultural, Refugees, Posttraumatic stress disorder, Factor analysis

## Abstract

**Background:**

Refugees are known to have high rates of post-traumatic stress disorder (PTSD). Although recent years have seen an increase in the number of refugees from Arabic speaking countries in the Middle East, no study so far has validated the construct of PTSD in an Arabic speaking sample of refugees.

**Methods:**

Responses to the Harvard Trauma Questionnaire (HTQ) were obtained from 409 Arabic-speaking refugees diagnosed with PTSD and undergoing treatment in Denmark. Confirmatory factor analysis was used to test and compare five alternative models.

**Results:**

All four- and five-factor models provided sufficient fit indices. However, a combination of excessively small clusters, and a case of mistranslation in the official Arabic translation of the HTQ, rendered results two of the models inadmissible. A post hoc analysis revealed that a simpler factor structure is supported, once local dependence is addressed.

**Conclusions:**

Overall, the construct of PTSD is supported in this sample of Arabic-speaking refugees. Apart from pursuing maximum fit, future studies may wish to test simpler, potentially more stable models, which allow a more informative analysis of individual items.

## Background

Post-traumatic stress disorder (PTSD) was introduced as a formal diagnosis in 1980 with the Diagnostic and Statistical Manual of Mental Disorders (*DSM-III*). Apart from its focus on an external etiological agent, a key characteristic of PTSD is the categorization of symptoms into clusters. In order to receive a diagnosis of PTSD, patients must display a number of persistent, trauma-related symptoms from each of these clusters (Table 1). A number of alternative structures have been suggested and tested, and recently the official factor structure was revised in the *DSM-5* [1].

**Table 1:**
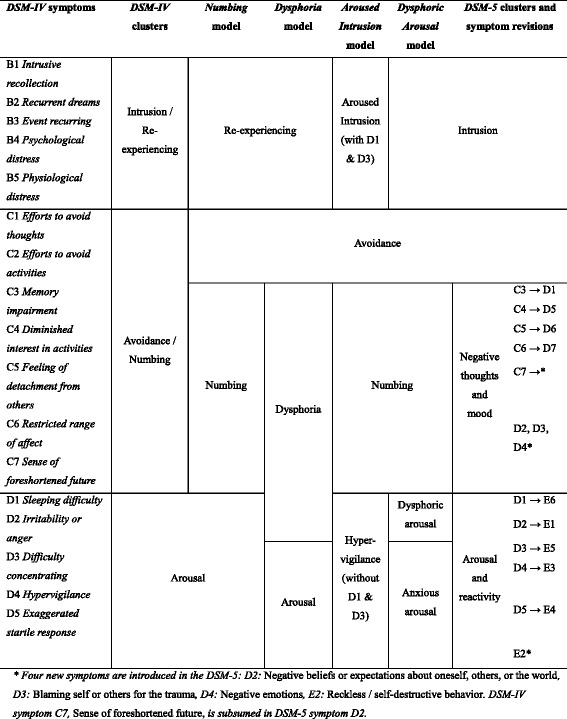
Overview of factor models of PTSD symptoms

An important challenge in establishing the factor structure of PTSD is testing it across populations characterised by different traumas and cultural backgrounds [44]. The PTSD symptom pattern of refugees may deviate from those of Western populations because of both cultural and war-related factors, as well as post-traumatic life circumstances. Traumatised refugees are typically characterised by an extensive trauma history, exposure to torture and rape, and often conflict-related death of family members. Eventually forced to flee, their social network is disrupted and their personal ambitions for the future, such as building a career and a family, are challenged. To cover the potentially wide range of distress relating to such extensive life disruptions, the assessment may benefit from a context sensitive framework, such as the Cultural Formulation Interview or the Adaptation and Development after Persecution and Trauma (ADAPT) model [2, 45]. At the same time, the symptom structure within the confines of the PTSD diagnosis may reflect the extent of such complexities in a more generic way.

The importance of understanding cultural differences in PTSD becomes particularly salient when one considers how PTSD is distributed across cultures. An epidemiological meta-analysis by Steel and colleagues estimates a 13 to 25 % prevalence of PTSD among refugees [48]. In the EU alone, the annual number of asylum applicants ranges from 200,000 to more than 1.2 million [12].

For refugee and non-refugee populations alike, the cluster structure of PTSD impacts the selection of cases. Based on a sample of 835 traumatic injury survivors, Forbes et al. [13] found that by following the *DSM-5* cluster structure in specifically requiring the presence of effortful avoidance, the number of PTSD cases was reduced by 26 %. On the other hand, such a decrease in prevalence may be compensated for when using the full *DSM-5* criteria, as it removes the requirement for *fear, helplessness, or horror* to have occurred right after the trauma. O’Donnell et al. [36], in a sample of 510 randomly selected injury patients, found that dropping this requirement from the *DSM-IV* resulted in a 25 % increase of cases. A study of West Papuan refugees did indeed find an almost equal number of *DSM-IV* and *DSM-5* cases, 12 and 13 % respectively [49]. Despite an apparent compensation in numbers, cases can shift dramatically between the two sets of criteria; O’Donnell et al. found that 22 participants met both sets of criteria, 12 only met those of *DSM-5* and eight only met those of *DSM-IV*. This indicates that a considerable portion of trauma affected patients have their diagnostic status determined by symptom cluster criteria. Furthermore, the symptoms of effortful avoidance may be less endorsed in a number of non-Western cultures [16]. Thus, we would expect the impact of the new structure to be particularly strong in these populations.

### The DSM models and proposed alternatives

The division of PTSD symptoms into three clusters in the *DSM-III* was originally based on expert opinion [43]. The model has since been tested using confirmatory factor analysis (CFA) in a number of studies, yielding no support [55]. The main criticism points to the categorization of symptoms of effortful avoidance (cognitive and emotional, as well as behavioural) with symptoms concerning numbing of general responsiveness, such as diminished interest and restricted range of affect. Alternative models thus delimit symptoms of effortful avoidance as a separate cluster, resulting in a better fit [55]. The *DSM-5* has been revised accordingly, resulting in four rather than three clusters. While the *DSM-5* also adds and changes a number of symptoms, the symptoms that most models disagree on remain mostly unchanged.

Within the *DSM-IV* framework, the three predominant PTSD models differ with respect to the placement of symptoms D1-D3, i.e. *difficulty sleeping*, *irritability*, and *difficulty concentrating*. Conceptually, the question is whether these symptoms (1) should be considered part of an anxious arousal construct (as in the *DSM*), (2) should be considered part of a depressed/dysphoric construct, or (3) warrant a separate factor [11]. This corresponds to three different models: The 4-factor Numbing model [21], the 4-factor Dysphoria model [46], and the 5-factor Dysphoric Arousal model [11]. As the 5-factor model is a relatively recent conception, to our knowledge it has not yet been tested in a refugee population. It has, however, been tested in at least three studies of non-Western, non-refugee populations, showing favourable results [4, 26, 53]. It has also generally been found to provide superior fit compared to alternative models [3].

### Previous studies with refugee populations

There is a clear underrepresentation of the Middle East in existing studies on the factorial structure of PTSD among refugees. Across a total of eight previous studies, the included refugee populations are made up of 4202 West and Central Africans, 682 Cambodians, 729 Burmese, 74 Vietnamese, 230 West Papuans, and a sample of 109 refugees from different countries in the Middle East [28, 38, 40, 42, 44, 47, 49, 51]. The study of Michalopoulos and colleagues [28] includes a sample of 974 Kurdish, non-refugee, torture survivors, but the authors were unable to compare the fit of competing models with this particular population (We suspect this may relate to a very low symptoms endorsement).

While the above studies mainly focus on the Numbing and Dysphoria models, the study by [40] introduces a third 4-factor model, which is the only model to modify the intrusion factor. Allegedly based on “the literature on posttraumatic stress among Africans and the authors’ clinical observations working with African trauma patients”, their *Aroused Intrusion* model modifies the Numbing model to have the symptoms insomnia (D1) and difficulty concentrating (D3) load on the intrusion factor. This results in what they term the *aroused intrusion* factor, while the would-be arousal factor, now consisting of only 3 items, is renamed the *hypervigilance* factor (Table 1)*.* This model showed slightly stronger support than the numbing model in the study by Rasmussen and colleagues. However, this was not replicated in the study by [51], where the Aroused Intrusion model showed the poorest fit of all the 4-factor models. We know of no other study to have tested the Aroused Intrusion model.

### Aims of this study

In this study, we used confirmatory factor analysis to test and compare five models of PTSD in a sample of 409 Arabic-speaking refugees undergoing PTSD treatment in Denmark. The DSM-IV-TR model and four subsidiary models were tested: The Numbing model, the Dysphoria model, the Aroused Intrusion model, and the Dysphoric Arousal model. Based on existing literature, we hypothesised that the 4- and 5-factor models would provide a better fit than the 3-factor DSM-IV model, and that one or more of these models would provide satisfactory fit. Apart from a confirmatory assessment of the overall fit of the models, we also aimed, through an exploratory approach, to provide information on specific sources of potential misfit across the models, e.g. related to individual items or pairs of items.

## Methods

### Subjects

All data for this study were collected at the Competence Centre for Transcultural Psychiatry (CTP) from 2008 to 2012 [10]. All respondents were Arabic-speaking refugees fulfilling the criteria for PTSD according to ICD-10 (F43.1) and undergoing psychiatric treatment at CTP. Patients fulfilling ICD-10 criteria for mental and behavioural disorders due to psychoactive substance use (F10-F19) or Schizophrenia, Schizotypal or delusional disorders (F20-F29) were excluded.

The sample consisted of a total of 409 subjects, of which 223 (55 %) were male and 186 (45 %) were female. The average time since arrival in Denmark was 15.3 years (*SD* = 6.3). Time since primary trauma was more than 20 years for 65 % of patients, more than 15 years for 81 %, more than 10 years for 91 %, and more than 5 years for 97 %. The main countries of origin were Iraq (65 %) and Lebanon (26 %). Of the total sample, 98.5 % had a diagnosis of Major Depression.

A representable subsample of the population (those undergoing treatment during 2008–2009) is described in more detail in [9]. The data collection procedure at CTP, as an integrated part of treatment, is described in [10].

### Instruments

The analysis is based on answers to an Arabic translation of the Harvard Trauma Questionnaire (HTQ; [31]). The HTQ has been widely used internationally to assess PTSD symptoms in refugees [19, 22, 30, 37, 41]. It was used in four of the previously mentioned six studies on the factor structure of PTSD in refugees [28, 38, 40, 44]. It contains four parts, ranging from assessment of life events to present symptoms, and from open-ended questions to four-category Likert items. Most parts are adapted to a specific cultural setting. The website of the Harvard Program in Refugee Trauma, as of this writing, lists six cultural versions of the Questionnaire [15].

The current study makes use of only the first 16 items of part 4 of the HTQ, which correspond to the symptoms of the *DSM-IV* criteria B, C, and D. The total of 16 items in the HTQ as opposed to 17 symptoms in the *DSM-IV* is the result of a compounding of *DSM* symptoms B4 and B5 into one item in the HTQ (Item 16: *Sudden emotional or physical reaction when reminded of the most hurtful or traumatic events*). Each item has four response options with a score from 1 to 4: *Not at all, a little bit, quite a bit,* and *extremely*.

The Arabic version of the HTQ used in this study was first tested by the Rehabilitation Council for Torture Victims and is referred to in the official manual for the Indochinese versions of the HTQ [32]. While the translational process for the Indochinese versions is described in both the manual and the original HTQ validation study [31], no such information is provided for the Arabic version. We know of no other widely distributed Arabic translation of the HTQ, and, to our knowledge, no previous study has reported on the factor structure of the Arabic translation of the HTQ.

### Procedure

All ratings for the current study were obtained at baseline. Patients would either read the questionnaire themselves or have it read to them by a translator. The latter option was used with illiterate patients, as well as patients with severe headaches or other physical conditions, which would otherwise prevent them from filling in the battery of questionnaires. The questionnaire was introduced to the patient by a medical doctor, who was also available during the administration to answer potential questions and to ensure that all items were completed.

All participants filling out the questionnaires have given informed consent. The involved trials have been conducted in accordance with the Helsinki Declaration and have been approved by the Danish National Committee on Research Ethics.

### Statistical analysis

Data Analysis was performed with the Mplus software (Version 7.31, [33]). We treated the HTQ items as ordinal variables, following arguments of [5], and based on indications of ceiling effects in the current data (see Table 2). A confirmatory factor analysis was conducted in order to assess the fit of the data to each of the 3-, 4- and 5-factor models. Reported goodness-of-fit indices consist of the comparative fit index (CFI), the Tucker-Lewis index (TLI), and the root mean square of approximation (RMSEA). An acceptable fit is indicated by CFI and TLI ≥0.90, and RMSEA ≤0.06 [17]. For nested models, we compared fit using a χ2 difference test [35]. For the comparison of non-nested models, we used Akaike Information Criterion (AIC) and Bayesian Information Criteria (BIC). As these are not available for categorical data with the weighted least squares means and variance adjusted estimator (WLSMV), they were obtained, where applicable, through a separate analysis with maximum likelihood estimation (MLR). For AIC and BIC smaller numbers indicate better fit and a difference greater than 10 is considered strong evidence [20].

**Table 2:** Mean scores and standard deviations (SD) for the Harvard Trauma Questionnaire

*DSM-IV* symptom	HTQ item	Average score	SD	Skewness	Kurtosis
B1 *Intrusive recollection*	1	3.45	.65	−0.95	3.48
B2 *Recurrent dreams*	3	3.45	.68	−1.08	3.80
B3 *Event recurring*	2	3.30	.74	−0.76	2.96
B4 & B5					
* Psychological & physiological distress* (combined into one item)	16	3.48	.65	−1.09	3.97
C1 *Efforts to avoid thoughts*	15	2.91	1.02	−0.58	2.20
C2 Efforts to avoid activities	11	3.21	.92	−0.96	2.98
C3 *Memory impairment*	12	2.10	1.07	0.41	1.82
C4 *Diminished interest in activities*	13	3.16	.87	−0.73	2.66
C5 *Feeling of detachment from others*	4	3.24	.88	−1.03	3.33
C6 *Restricted range of affect*	5	2.49	1.13	−0.06	1.60
C7 *Sense of foreshortened future*	14	3.50	.81	−1.60	4.84
D1 *Sleeping difficulty*	8	3.57	.62	−1.27	4.05
D2 *Irritability or anger*	10	3.32	.80	−0.99	3.30
D3 *Difficulty concentrating*	7	3.48	.68	−1.12	3.74
D4 *Hypervigilance*	9	3.24	.84	−1.04	3.59
D5 *Exaggerated startle response*	6	3.35	.79	−1.09	3.62

After testing the fit of the standard models, a post hoc analysis was carried out based on modification indices (MI) and content analysis. MIs estimate how much the fit of a model would improve by relaxing individual parameters of that model, and they may supplement global estimates of fit by locating specific sources of misfit [7, 8]. Along with the MI, the fully standardized expected parameter change (EPC) was inspected, to evaluate expected changes in particular loadings or residual correlations due to suggested modifications. In cases of a particularly high MI and corresponding EPC, we evaluated whether the suggested specification made sense from a clinical perspective or could be related to an inadequate translation in the Arabic HTQ. In such cases we tested if re-specification had substantial impact on the relative fit of the models.

As a last step, we evaluated if the models could be simplified, i.e. if any parameters were statistically non-significant. We did this by collapsing those factors that displayed correlations near 1, and finally by testing a unidimensional model.

## Results

Item means and standard deviations for the responses to the HTQ are presented in Table 2. The mean scale score is 51.1, corresponding to a mean item score of 3.2 (*SD* = 0.37, range: 1.9–4). Fit statistics for the competing CFA models are presented in Table 3. Results show that the 3-factor *DSM-IV* model provides a poor fit to the data while all 4- and 5-factor models show acceptable CFI, TLI and RMSEA. The Dysphoria model and the Dysphoric Arousal model both feature a standardized inter-factor correlation exceeding 1, in both cases involving the two-item anxious arousal factor (Table 4). This indicates that the models in each case cannot distinguish the two factors, effectively rendering the resulting fit indices inadmissible [34]. Thus, for the predefined models, only the Numbing and Aroused Intrusion models could be evaluated. Comparing these with a separate analysis using MLR estimation indicated superior fit of the Aroused Intrusion model, AIC = 13330.095 and BIC = 13611.055, over the Numbing model, AIC = 13353.805 and BIC = 13634.765.

**Table 3:** Fit Statistics for the tested models

Model	χ^2^ (*df*)	RMSEA	CFI	TLI
*DSM-IV*	275.298 (101)	0.065	0.889	0.868
Numbing (King et al.)	206.088 (98)	0.052	0.931	0.916
Dysphoria (Simms et al.)	225.694 (98)^a^	0.056^a^	0.918^a^	0.900^a^
Dysphoric Arousal (Elhai et al.)	199.370 (94)^a^	0.052^a^	0.933^a^	0.914^a^
Aroused Intrusion (Rasmussen et al.)	176.765 (98)	0.044	0.950	0.938

*Note: df* degrees of freedom, *RMSEA* root mean square error or approximation, *SRMR* standardized root mean square residual, *AIC* Akaike information criterion, *CFI* comparative fit index, *TLI* Tucker-Lewis Index, *BIC* Bayesian information criterion; *DSM* diagnostic and statistical manual of mental disorders. All χ^2^ values are statistically significant at *p* <.001

^a^Results for the Dysphoric Arousal model and Dysphoria model are inadmissible

**Table 4:** Factor correlations for the Numbing, Dysphoric Arousal and Aroused Intrusion models of PTSD

Numbing model	Intrusion	Avoidance	Numbing	Arousal	
Intrusion	1				
Avoidance	.30	1			
Numbing	.63	.32	1		
Arousal	.77	.28	.76	1	
Dysphoric Arousal model	Intrusion	Avoidance	Numbing	D.A.	A.A.
Intrusion	1				
Avoidance	.30	1			
Numbing	.63	.32	1		
D.A.	.81	.30	.81	1	
A.A.	.91	.33	.88	1.33	1
Aroused Intrusion model	A.I.	Avoidance	Numbing	Hypervigilance	
A.I.	1				
Avoidance	.31	1			
Numbing	.70	.32	1		
Hypervigilance	.66	.25	.71	1	

*D.A* dysphoric arousal, *A.A* anxious arousal, *A.I* aroused intrusion

### Post hoc analysis

Modification indices (MI) suggested for all models to have item D2, (*Feeling irritable or having outbursts of anger)*, correlate freely with item D5, (*Exaggerated startle response)*. Across the 4- and 5-factor models, the size of the MI ranged from 14.9 in the Aroused Intrusion model to 45.0 in the Dysphoria model, and the fully standardised expected parameter change (EPC) ranged from 0.31 to 0.34. In other words, if allowing item D2 and D5 to correlate freely, the χ^2^ value of each model was estimated to drop between 14.9 and 45.0 points and the residual correlation was estimated to rise from zero to somewhere between 0.31 and 0.34. A post hoc content analysis of the adaptation to Arabic, which is elaborated in the Discussion section, provided support for an unintended content overlap between the two items. Removing item D2 from the model was the only modification which would allow a comparison of all models with only one, shared modification. Doing so resolved the problem of correlations above 1 in both the Dysphoric Arousal model and the Dysphoria model. Fit increased across all models, yielding virtually identical fit indices for the Numbing, Aroused Intrusion, and Dysphoric Arousal models (Numbing model: CFI = 0.956, TLI = 0.945, RMSEA = 0.041; Dysphoria model: CFI = 0.940, TLI = 0.925, RMSEA = 0.048; Dysphoric Arousal model: CFI = 0.959, TLI = 0.946, RMSEA = 0.041; Aroused Intrusion model: CFI = 0.956, TLI = 0.944, RMSEA = 0.041). There was no significant difference between the fit of the Dysphoric Arousal model and the Numbing model, χ^2^ difference(4) = 8.288, *n.s.* Obtaining AIC and BIC through a consecutive estimation with Maximum Likelihood gave equal support for the Numbing model (AIC 12564.488, BIC 12829.393) and Aroused Intrusion model (AIC 12565.252, BIC 12830.157). The Dysphoric Arousal model displayed a similar fit in terms of AIC, but received substantially less support in terms of BIC, reflecting a penalty for excessive complexity (AIC 12565.467, BIC 12846.427). The Dysphoria displayed the poorest fit (AIC 12584.845, BIC 12849.750).

Modification indices also suggested for all models to have item B1, *Recurrent thoughts or memories of the most hurtful or terrifying event*, and B3, *Feeling as though the event is happening again*, correlate freely. The size of the MI ranged from 19.0 to 23.2, and EPC was 0.27.

High correlations were observed between the factors of intrusion, aroused intrusion, arousal, dysphoric arousal and anxious arousal, ranging from 0.84 to 0.99 in the modified models. We tested whether reducing the number of factors would decrease fit substantially when the above residual correlations were included in the models. Only the Numbing and Aroused Intrusion models were used as null models, allowing all 16 items to be used. Items D2 and D5, and B1 and B3 were allowed to correlate freely. For the Numbing model, collapsing the arousal factor with the intrusion factor did not result in a significant decrease of fit, χ^2^ change(3) = 4.866, *n.s.* For the Aroused Intrusion model, collapsing the hypervigilance factor with the numbing factor did not result in a significant decrease of fit, χ^2^ change (3) = 7.096, *n.s.* All remaining collapses resulted in a significant decrease in fit.

Finally, a single factor model was formed by collapsing all clusters and allowing free correlation between the two avoidance items, C1 and C3, in addition to the previously modelled errors for item pairs D2 & D5, and B1 & B3. Fit indices for this model were: CFI = 0.954, TLI = 0.945, RMSEA = 0.042.

Throughout the analysis, it was generally observed that the loading of symptom C3, *memory impairment*, was particularly low. Memory impairment consistently loaded below 0.2, while the next weakest loading, that of symptom C6, *restricted range of affect*, loaded above 0.6. Neither modification indices or item correlations indicated that memory impairment would load significantly higher on any other factor. The highest factor loading for the single factor model was that of symptom C5, *feeling of detachment from others,* followed by C7, *sense of foreshortened future.*

## Discussion

The present study is, to our knowledge, the first to assess the factorial structure of PTSD in an Arabic-speaking population of refugees. Results replicate previous findings that a 4-factor model with a separate avoidance factor provides a better fit than the *DSM-IV* model. In this regard, the study adds cross-cultural support for the decision to place symptoms of effortful avoidance in a separate cluster in the *DSM-5*.

Two models could not be properly estimated, namely those separating items D2 and D5. Looking at the original, English, wording in the HTQ, symptom D5 is formulated: “Feeling jumpy, easily startled.” Asking two separate Arabic translators to back translate this item, they noted a connotation of “flare up” (سرعة الهيجان). This constitutes an unintended content overlap with item D2, *Feeling irritable or having outbursts of anger*. As noted, removing item D2 was the only solution to this problem, which could be applied across all of the models. With this modification, CFI, TLI and RMSEA were virtually identical across the Numbing-, Dysphoric Arousal-, and Aroused Intrusion model. That the 5-factor model did not offer a significantly better fit than the 4-factor Numbing model supports the latter as a more parsimonious model. This was also reflected in the BIC, which had a steeper penalty of model complexity and thus provided strong evidence against the 5-factor model. We cannot know for certain how much the unintended overlap of items D2 and D5, as well as our removal of item D2, influences these results. Removing item D5, rather than D2, would perhaps do more justice to the Dysphoria and Dysphoric Arousal models as item D5 introduces a component of anger in the anxious arousal cluster. But with only two items on the anxious arousal factor, item D5 was indispensable.

Another notable error correlation concerned symptoms B1, “Recurrent thoughts or memories of the most hurtful or terrifying event”, and B3*,* “Feeling as though the event is happening again”*.* There was no indication that the content overlap was any larger in the Arabic translation than in the original, English version. Rather than being an artefact, we believe this result reflects a particularly close relation between these two symptoms. At least in these authors’ clinical experience, trauma patients can easily progress from cued recall, e.g. when asked about circumstances surrounding the trauma, to gradually dissociate in their sensory experience. A higher average endorsement of item B1 (*M* = 3.45) over item B3 (*M* = 3.30) provides some support for the idea that recall offers a prerequisite for re-experiencing. Future studies may wish to report if such a substantive error correlation is replicated.

Unspecified residual correlations may have a crucial impact on the evaluation of alternative models. When MI and EPC suggest freeing up the correlation between B1 and B3, it means that item B1 and B3 had a higher correlation than the shared construct of intrusion could properly account for. This is reflected in the correlations among the residuals, which for items B1 and B3 is estimated to be 0.27 for optimal fit. Restricting this correlation to zero introduces strain on the intrusion factor, which is forced to account for all of the shared variance of B1 and B3. The loadings of the involved item may become inflated in this situation, and/or remaining loadings on the factor may become deflated [8]. The strain may influence models differently; For the items D2 and D5, the MIs indicated that the best fitting baseline model was least influenced, while the poorest fitting 4-factor model was influenced the most. A comparison of models based exclusively on global fit may thus favour a model because it is less influenced by unintended local sources of strain. Researchers in the field of transcultural psychiatry should be particularly alert to such potential methods effects as there is more to go wrong upstream in cross-cultural assessment, including translation and cultural adaptation of instruments.

Another methodological issue that may easily influence the validity of cross-cultural CFA-studies is the minimal size of some of the theorised factors. Fewer items in a factor will generally challenge replicability of a given factor structure [24, 50]. In this regard, the original *DSM* model provided a relatively even distribution of items across clusters. Although the separation of avoidance symptoms in the *DSM-5* is informed by a vast number of studies reporting superior fit of this configuration, we believe it deserves further psychometric scrutiny on two accounts. First, to our knowledge, no prior study has tested the Numbing model against a *DSM-IV* model with error terms between the two avoidance items. If such a test does not favour the 4-factor Numbing model, the psychometric support for a separate avoidance factor becomes less evident. Second, if avoidance is found to constitute an independent latent trait, then that trait should arguably receive full content coverage. If other factors are covered by five to seven symptoms, then, from a psychometric perspective, it is not clear why avoidance is only characterized by two symptoms.

Similar points can be raised with regard to the two-item anxious arousal factor. But given that it is not unequivocally supported, and given the introduction of new symptoms in the *DSM-5*, future directions for testing this factor are less clear. The arousal cluster sees one new symptom in the *DSM-5*, *reckless behaviour*, which could potentially help stabilise either the dysphoric arousal or anxious arousal factor. According to [14], it was included in the *DSM-5* because it is seen as an important symptom in traumatised adolescents. Initial factor studies of the *DSM-5*, however, indicate poor loadings of this item [25, 29]. If small factors persist, we would urge future cross-cultural CFA studies to exert caution in the evaluation of these.

The single-factor model with three error correlations showed good fit indices. One should always be highly cautious when interpreting fit indices based on modelled fit residuals, as they are likely to represent overfitting to the particular sample [18, 23]. However, the result indicates that in this particular sample, the HTQ can be treated as a unidimensional scale. This implies that an analysis based on item response theory could provide more information about how individual items contribute to the scale as a whole, as well as how they contribute differently across gender and age. Future studies may test whether a general factor PTSD model, which allows residual correlations between item B1 & B3, and between the avoidance items, shows acceptable fit in other samples of refugees.

A number of observations regarding individual items are worth noting. Symptom C3, trauma related amnesia, consistently loaded below 0.2 and thus make a poor contribution to the construct of PTSD in this sample. A number of previous cross-cultural studies also reported this as the weakest loading item [22, 26, 38, 44, 53]. It is perhaps the most disputed symptom of PTSD and critics question whether dissociative amnesia is a likely, or even possible, result from traumatic experiences [27, 39]. Symptom C6, inability to feel emotions, also displayed a relatively low endorsement and loading. According to a number of clinicians and interpreters working with the present sample, it is the item most frequently inquired about. We believe that an ‘inability to feel positive emotions’, in accordance with the *DSM-5*, will be a much easier concept to convey across cultures, particularly in a questionnaire form.

Regarding avoidance symptoms, patients will often report verbally that they try intensely to avoid thoughts and feelings of the traumatic events but repeatedly fail in these efforts. Asking patients to rate their distress from any attempted avoidance of thoughts and feelings, rather than only successful avoidance, may afford a more valid assessment in this population. Similarly, regarding the avoidance of activities, some patients express a perceived comfort in complete social isolation, while being distressed from social demands. Although they may have particularly fearful reactions to domain specific situations, such as seeing uniformed men, they will often report being uncomfortable around strangers in general. In this context, it may be beneficial to assess social isolation with specific and separate reference to depression and anxiety, e.g. “Avoid leaving my home because I expect other people to look down on me” and “Avoid leaving my home because I expect to witness or become a victim of violence”.

As noted, the DSM-5 introduces a number of new symptoms, which are not included in the HTQ, and consequently not covered in this study. The DSM-5 was officially introduced in 2013, and other PTSD scales have been updated to meet the new content, e.g. the Posttraumatic Stress Disorder Checklist [6] and the Clinician-Administered PTSD Scale (PCL-5; [54]). However, we found no indication that revisions of the HTQ are planned. To promote transparency and standardization, future studies may wish to adapt DSM-5 and ICD-11 items from existing questionnaires, such as the PCL-5. The new DSM-5 symptoms mainly concern the numbing factor, which now contains seven symptoms and is named ‘negative alterations in cognitions and mood’ (Table 1). We note that the Numbing cluster is the only large cluster, which is not divided in any of the models. Thus, in terms of factor structure, it could be considered the cluster least likely to be affected by additional items. The impact of the new arousal item, ‘reckless or self-destructive behavior’, is more difficult to estimate. As already noted, it has received low endorsement in initial studies, and, based on clinical experience, we would expect this to be the case in the population tested in the current study. We would encourage future studies to report whether the contribution of this particular item is clear, and when this is not the case, to explore alternative solutions.

From a clinical perspective, the current study supports the construct of PTSD in Arabic-speaking refugees, and as such supports the use of interventions targeting PTSD. Still, it is important to consider the influence of comorbid depression in a sample as chronic as this. Not only is the comorbidity of depression almost absolute, also symptom C5, “*Feeling detached or withdrawn from people*”, and C7, “*Feeling as though you don’t have a future”*, displayed the highest loadings on the single factor PTSD model. These symptoms will likely need addressing from the beginning of therapy, in order to provide a motivational platform for deliberate cognitive and behavioural exercises. One possible way to pursue this is through working with personal values, both rediscovering old values and adapting them to the new life circumstances, e.g. as described in Acceptance and Commitment Therapy [52]. Facilitating social contact in a welcoming environment, with no stigmatizing associations within the culture of the patient, may also be important for patients who have become excessively isolated.

## Conclusions

In conclusion, results of this study indicate that the construct of PTSD is valid among Arabic speaking refugees. Among the alternative models, the Numbing model and the Aroused Intrusion models received most support. Methodologically, the study points to a number of potential improvements in studies of the factor structure of PTSD across cultures. Firstly, future studies may report not only on the global fit, but also on evidence of local sources of misfit. Secondly, studies may include tests of simpler models, such as a two factor model with error terms between item B1 and B3, and a single factor model with additional error terms between the avoidance items. Finally, we would urge researcher who use translated instruments to always state the origin of the translations.

## Abbreviations

AIC, Akaike Information Criterion; BIC, Bayesian Information Criteria; CFA, confirmatory factor analysis; CFI, comparative fit index; CTP, Competence Centre for Transcultural Psychiatry; df, degrees of freedom; DSM, diagnostic and statistical manual of mental disorders; EPC, expected parameter change (fully standardized); HTQ, Harvard Trauma Questionnaire; ICD, international classification of diseases; MI, modification indices; MLR, maximum likelihood (robust); PCL-5, the Clinician-Administered PTSD Scale, DSM-5 version; PTSD, post-traumatic stress disorder; RMSEA, root mean square of approximation; SD, standard deviation; TLI, Tucker-Lewis index; WLSMV, weighted least squares means and variance adjusted estimator
